# Management of Renal Artery Aneurysms and an Arteriovenous Shunt in a Hemophilic Patient: A Case Report

**DOI:** 10.7759/cureus.55218

**Published:** 2024-02-29

**Authors:** Yehya Tlaiss, Aziz M Najjar, Mohamad Tlais, Peter Noun, Imad Ghantous

**Affiliations:** 1 Urology, University of Balamand, Beirut, LBN; 2 Urology, Hôpitaux Universitaires Pitié Salpêtrière, Paris, FRA; 3 Hematology and Medical Oncology, Saint George Hospital University Medical Center, Beirut, LBN; 4 Urology, Saint George Hospital University Medical Center, Beirut, LBN

**Keywords:** endovascular intervention, renal artery aneurysms, renal avm embolisation, renal arteriovenous shunt, hemophilia-a, recurrent hematuria

## Abstract

This case report details the management of a 66-year-old male with hemophilia A (HA) who presented with recurrent hematuria, and was found to have renal artery aneurysms and was subsequently diagnosed with a renal arteriovenous (AV) shunt. While the primary focus centers on the successful endovascular coil embolization of renal artery aneurysms, the concomitant presence of the AV shunt accentuates the significance of this case. Imaging techniques were crucial in the discovery of renal aneurysms and the diagnosis of the AV shunt malformation of the renal artery. This included an ultrasound, CT-angiography and digital subtraction angiography. The treatment approach employed prioritized endovascular coil embolization for its efficacy and reduced morbidity. Following the initial successful embolization, the identification of the AV shunt during subsequent embolization led to its targeted treatment. The case was also complicated by acute prostatitis that was treated medically. The patient’s HA required careful administration of coagulation factor replacement therapy to control bleeding throughout the process. This case highlights the importance of reporting on the management of rare and complex pathologies to better understand and guide future treatments, especially involving this rare combination of renal AV shunts and hemophilia A.

## Introduction

Hemophilia is a congenital X-linked recessive bleeding disorder. Hemophilia A (HA) affects around 1 in every 5000 live male births compared to hemophilia B (HB) affecting 1 in 25,000 live male births [1]. People with HA have low levels of factor VIII (FVIII) and it can be symptomatic even in female carriers, which is due to the random X-chromosome inactivation during embryonic life (also known as the Lyonization phenomenon) [1]. The severity of HA is classified, based on the clotting FVIII level in the blood, into three stages: mild, moderate and severe. The concentration of functional FVIII in the plasma is more than 5% in mild cases, 1%-5% in moderate and less than 1% in severe [2]. Patients with HA typically show signs of bleeding as a clinical presentation that is more prominent in patients with severe hemophilia who are at the highest risk [3]. Clotting factor administration is the treatment of choice in hemophilic patients. Depending on the severity, type of bleed, and procedure, the dosage and regimen of the clotting factor are adjusted [3]. Before any surgical intervention, it is crucial that a factor substitution plan is made [4]. Renal artery aneurysm is a medical condition in which the renal artery bulges or is abnormally dilated. These aneurysms can develop in any part of the renal artery, which branches off the abdominal aorta, and vary in size considerably. Although most renal artery aneurysms are small and asymptomatic, larger ones or those that are growing rapidly can cause severe symptoms such as hematuria [4]. On the other hand, renal arteriovenous (AV) shunt is a rare pathologic condition where an abnormal connection or pathway exists between an artery and a vein [5]. This means that the blood flows from an artery into a vein without going through the capillary network, which bypasses the normal circulation. When this occurs in the kidney, it can cause complications such as renal hypertension, massive hematuria, retroperitoneal hemorrhage, pain and congestive heart failure [5].

AV shunts are divided into two categories: traumatic and non-traumatic. The imaging angioarchitecture of these AV shunts allows for the classification of these malformations and the selection of treatment and choice of embolic coils or plugs based on the cause (traumatic vs. non-traumatic). Both the detection and diagnosis of renal aneurysms and AV shunts are usually made by CT-angiography (CTA) that is followed by endovascular treatment as the method of choice [5]. For proper treatment, successful transcatheter embolization is required and complete occlusion of the shunted vessel is needed, to prevent emboli from migrating and preserve normal arterial branches. As in the literature, there have been very few reports of cases involving the embolization of arterial aneurysms in hemophilic patients, such as the case reported by Janicka-Kupra et al., cases of embolization for renal AV shunts have been even more scarce [6].

## Case presentation

**Figure 1:**
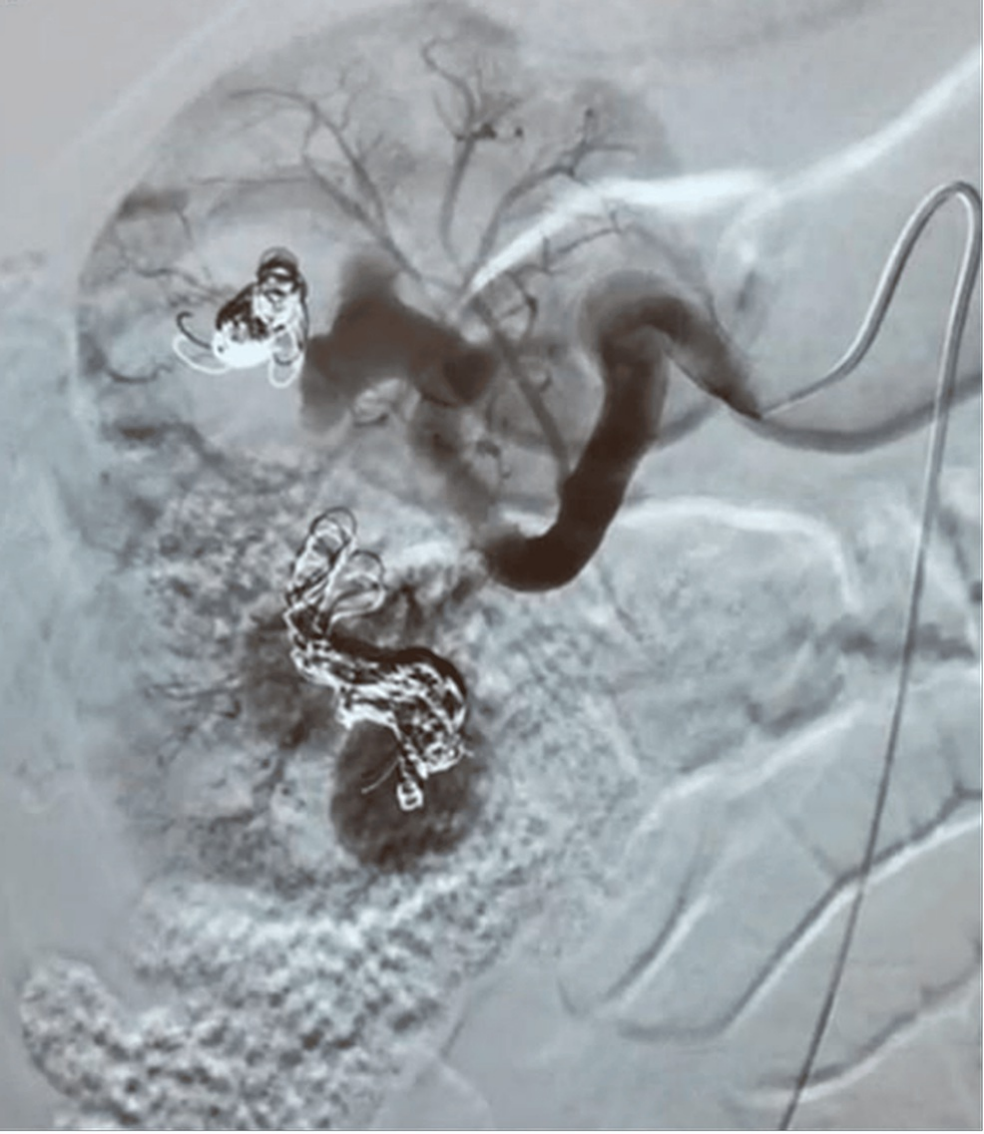
A digital subtraction angiogram showing coiling of right renal vessels

**Figure 2:**
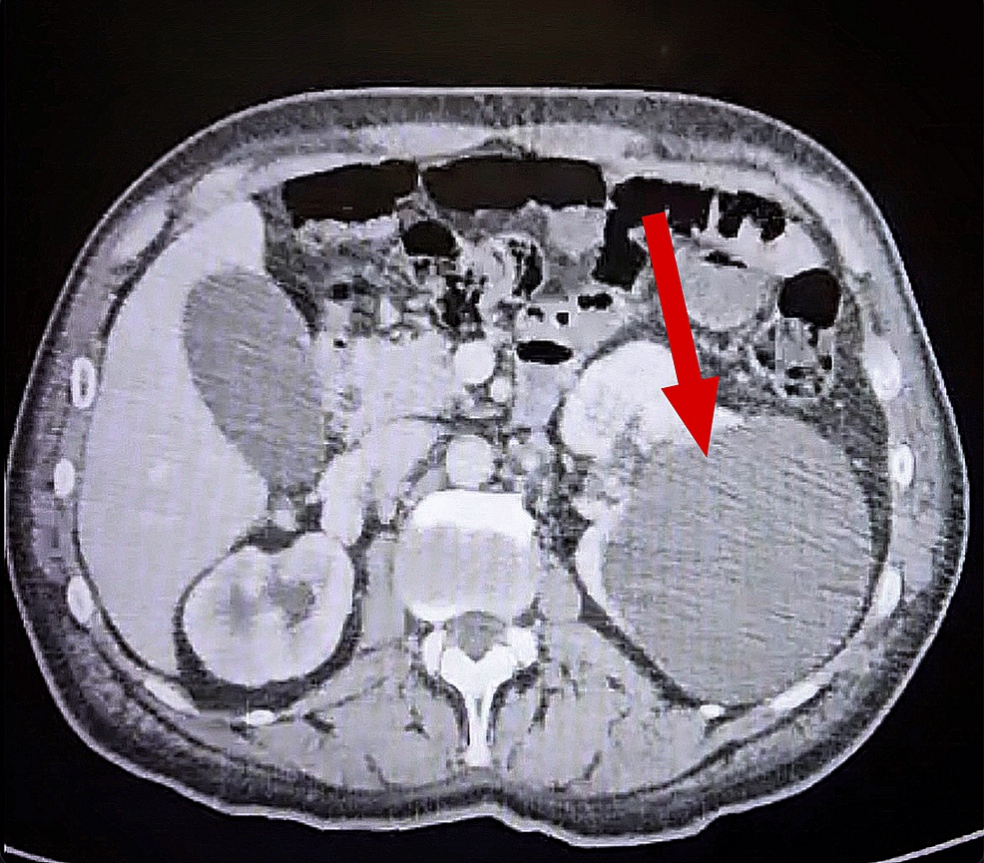
CT showing hematoma in the upper pole of the right kidney

A 66-year-old male patient with a history of hypertension and hemophilia A on factor VIII treatment presented with hematuria of 10-day duration. Initially, the patient noticed blood clots coming out while urinating; he also noticed that it would occur once yearly in the past due to his hemophilia, so he waited for it to resolve on its own. There was no fever or chills, no nausea or vomiting, and no urgency, frequency, nocturia or intermittency. Furthermore, the urinary stream remained normal despite blood clots. Ultrasound (US) of the pelvis and abdomen had been done four days prior to his presentation, showing the right kidney to be 40 cm in size along the axis with limited cortical thickness along with a large 11-cm cyst in the left kidney and a 3-mm formation hanging from the posterior wall of the bladder. The patient presented to the ER as recommended by his physician. Lab tests showed Hb at 8.5 g/dL, along with a creatinine level of 0.9 mg/dL and a high RBC count in the urinalysis. The patient was diagnosed with pelvicalyceal bleed, and was admitted for further management of his situation, along with FVIII inhibitor bypassing activity (FEIBA) administration. FEIBA is an anti-inhibitor coagulant complex used in the control and prevention of bleeding episodes in patients with hemophilia A [2]. As an inpatient, medical treatment was given, and right kidney embolization was done. During this first embolization, the initial right renal digital subtraction angiogram revealed markedly enlarged aneurysms arising in the middle and lower poles of the right kidney draining into large renal veins. Coil embolization was performed using multiple detachable coils and 0.018-inch coils (Figure 1). This was followed by an injection of dehydrated alcohol and gel foam in view of the absence of remaining coils. At the end of the procedure, there was near complete obliteration of the aneurysms and marked stasis of contrast in the segmental renal arteries. Since there were not enough coils to embolize both aneurysms, the patient was set to return in one month for repeat embolization even though there was marked stasis of contrast in the aneurysms and feeding arteries. Post-operatively, his hematuria improved, and the patient was discharged, though he presented three days later with residual hematuria, at which time he was advised to increase his fluid intake and was discharged again. The patient presented again three days later with worsening hematuria, and the CT-angiography done showed dilatation of the intrarenal arteries of the upper pole of the right kidney related to vascular malformation, along with aneurysmal dilatation of the middle third of the right renal artery. This malformation was consistent with a renal AV shunt. No thrombus was noted. Hematoma of the upper pole of the right kidney with no evidence of active bleeding was also present (Figure 2), along with arterial malformation of the upper pole of the left kidney. Pseudo-aneurysm of the right femoral vein was also noted. No active bleeding in the abdomen and pelvis, or any other change, was present. FEIBA was switched to NovoSeven at the time, and the patient was further monitored for hematuria. NovoSeven is an injectable coagulation FVIII recombinant.

**Figure 3:**
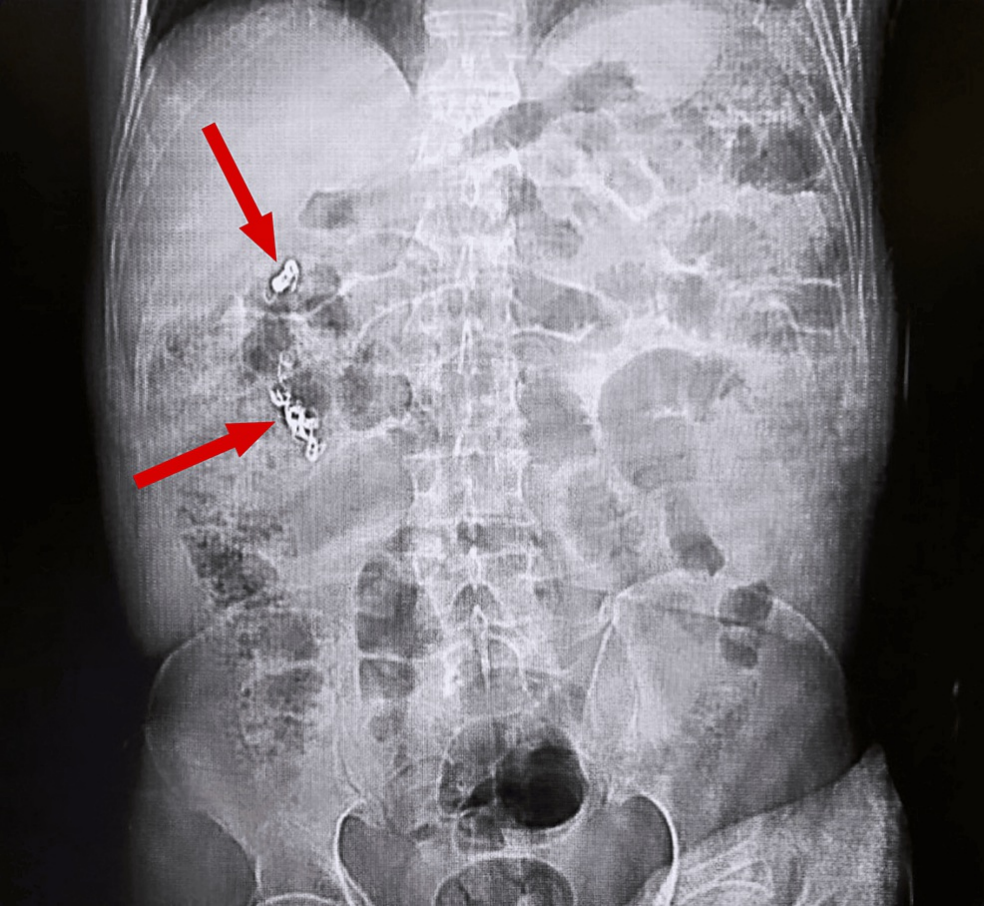
A KUB radiograph showing right renal vessel coils KUB, kidney, ureter and bladder

**Figure 4:**
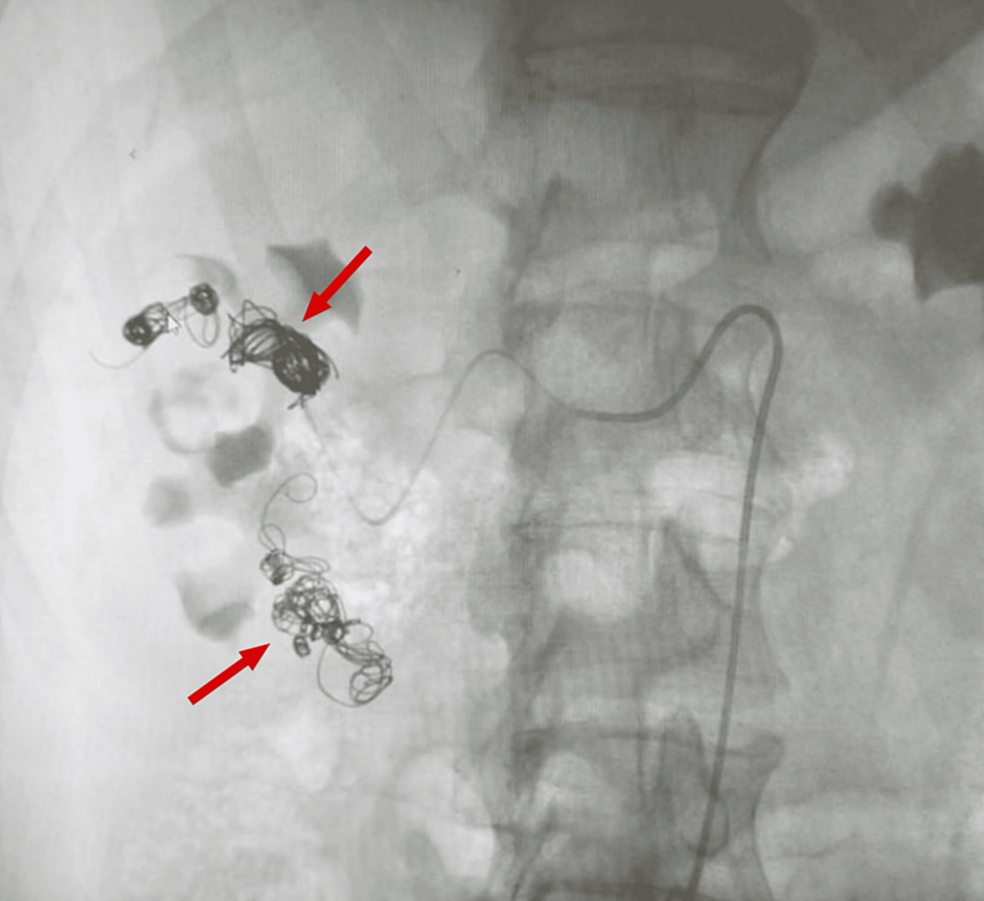
A KUB radiograph showing right renal vessel coils KUB, kidney, ureter and bladder

After three weeks, the patient was presented to our care, at which point coiling of right renal vascular malformations, the AV shunt, was done by an interventional radiologist (Figures 3, 4). During this second embolization, US evaluation of the right groin revealed a partially thrombosed hematoma with no connection to the right common femoral artery. Contrast injection revealed a fusiform aneurysm arising from the upper segmental artery taking the shape of the upper anterior calyx. The aneurysm was embolized using multiple detachable micro-coils. A follow-up contrast injection revealed no residual filling of the aneurysm with preferential flow of contrast to the rest of the kidney. The final arteriogram revealed a saccular aneurysm arising from the middle aspect of the main right renal artery. Multiple attempts made to stabilize the catheter in order to place a stent through the aneurysm failed. In addition, a total of 4000 units of thrombin were injected in the right groin hematoma that was seen to be immediately thrombosing. The initial arteriogram revealed a fusiform aneurysm arising from the upper segmental branch of the kidney taking the shape of the calyx. Post-operatively, there was no residual filling of the aneurysm, thanks to the targeted embolization performed on the AV shunt. After the embolization, the patient’s stay was complicated by acute prostatitis, for which he took antibiotics and was finally discharged on FEIBA.

## Discussion

In this report, we have discussed the case of a male patient with hemophilia A presenting with recurrent hematuria, found to have renal artery aneurysms and an AV shunt that was managed with endovascular coil embolization and FEIBA infusion. This case highlights an unusual hematuria presentation that includes an AV shunt malformation in the renal artery accompanied by HA that is rare in itself. Typical patients with AV shunts usually present with hematuria, hypertension or no symptoms at all [7]. The role of imaging in the detection of renal vascular malformations and AV shunts specifically is very important. A Doppler US can raise the suspicion of the existence of a renal AV shunt, but the use of angiography confirms it definitively. CTA is the standard criterion when it comes to the final diagnosis. However, digital subtraction angiography (DSA) is reserved for when more detail is intended and for cases where less invasive treatment is needed [5]. In our case, the patient underwent an abdominal pelvic US scan that showed abnormal findings. Hence, CT and DSA were done to confirm the diagnosis. The gold standard for treatment in such a case is endovascular embolization especially since hematuria was recurrent and required a total of two embolization procedures [7]. Endovascular techniques have been shown to have high efficacy and lower morbidity than old techniques like open surgical repair. However, with our patient having congenital hemophilia A and thus a high risk of major bleeding, embolization was the best treatment option. Embolization is an endovascular procedure shown to reduce blood loss in minimally invasive procedures [8]. Additionally, it has shown lesser needs for blood transfusions and shorter hospital stays on average [8]. Several studies have shown the importance of adding coagulation factor FVIII before, during and after surgery in hemophilic patients [9-11]. In our case, the patient received NovoSeven three weeks prior to his second embolization and was discharged on FEIBA. FEIBA was also given prior to his first embolization. Studies have shown that FEIBA limits post-surgical bleeding incidents significantly, even though coagulation factor VIII remains more effective [12]. A limited body of literature addresses this dual pathology, an AV shunt of the renal artery and hemophilia A, highlighting the significance of comprehensive case reporting. As mentioned earlier, major complications were noted, such as acute prostatitis, hematoma of the upper pole of the right kidney and recurrent hematuria that required re-embolization.

## Conclusions

In conclusion, this case report presents a unique case of a 66-year-old male with hemophilia A and recurrent hematuria who was found to have renal artery aneurysms and subsequently an AV shunt. Amidst the backdrop of endovascular coil embolization for renal artery aneurysms, the identification and management of the renal AV shunt highlights the complexity of this case. Imaging techniques were essential in the diagnosis of the AV shunt, as US, CTA and DSA were used. The treatment of choice was endovascular coil embolization for its lower morbidity and effectiveness. Additionally, the risk of major bleeding was high in our patient with hemophilia A, which necessitated careful management using coagulation factor replacement therapy to prevent and control bleeding episodes. Embolization was done twice in this case and the patient experienced some complications throughout, such as acute prostatitis, hematoma of the upper pole of the kidney and recurrent hematuria, which required the second embolization. This case emphasizes the necessity of scrutinizing and documenting rare intricate pathologies, to improve the understanding of such cases and guide future treatment strategies.
